# Short-Term Creep Prediction Model for Composite Geomembranes with Varying Film Thicknesses

**DOI:** 10.3390/ma19122622

**Published:** 2026-06-18

**Authors:** Yufan Hao, Xiaodong Wang, Sheng Feng, Jing Ma, Wu Yang, Shuhan Ma

**Affiliations:** 1 College of Hydraulic and Civil Engineering, Xinjiang Agricultural University, Urumqi 830052, China; haoyufan0909@163.com (Y.H.);; 2 Technical Research Center of Xinjiang Hydro-Geotechnical and Structural Engineering, Urumqi 830052, China; 3 Xinjiang Water Conservancy Development Investment (Group) Co., Ltd., Urumqi 830092, China; 4 Key Laboratory of Dam Construction Safety and Disaster Prevention of Xinjiang Production and Construction Corps, Urumqi 830004, China

**Keywords:** composite geomembrane, creep behavior, logarithmic function model, short-term prediction model

## Abstract

Composite geomembranes (GMs) are widely used in seepage projects, where their long-term deformation properties are critical for structural safety and stability. This study conducted a 90-day creep test to compare the deformation behavior of composite GMs with varying thicknesses. Based on the long-term creep data of the composite GMs, the average values of the model parameters and the variation rules of the coefficients of variation were analyzed. When the coefficients of variation for the creep exponent a and initial strain value b were both below 10%, a short-term empirical creep model across different thickness levels was established. The accuracy and applicability of the model were analyzed by comparing its results with the measured values. The results show that the long-term deformation behavior of the composite GMs across various thicknesses aligns with a logarithmic function containing thickness-dependent coefficients. Additionally, increasing film thickness leads to reduction in the final stabilized strain. The proposed short-term model based on experimental data demonstrated reasonable agreement between the model’s 72 h fitted data and the experimental measurements. Consequently, this model may serve as a useful empirical method for predicting the long-term creep deformation of composite GMs.

## 1. Introduction

Composite geomembranes (GMs), which integrate the properties of geotextiles and GMs, are widely used in seepage control projects in landfills and reservoirs [1,2,3,4,5]. As polymer-based anti-seepage materials, composite GMs exhibit remarkable rheological properties [6]. In practical engineering applications, composite GMs undergo creep deformation under long-term stress [7,8], which may weaken material strength or induce excessive deformation of the anti-seepage structure, thereby compromising the project safety. Accordingly, the creep and long-term stability performance of the composite GM are critical factors in determining whether it can function effectively in engineering applications over an extended period.

Creep performance is a critical durability property used to assess material service life. Currently, creep tests on composite GMs are typically conducted using two primary methods. The first is the standardized creep test, wherein materials are exposed to long-term loads under constant temperature and humidity conditions to thoroughly investigate their creep behavior. This test offers an accurate representation of the actual changes in the material and captures its deformation performance in detail [9,10,11,12,13]. However, the time required for the test is substantial, usually exceeding 1000 h, which limits its suitability for timely engineering design and rapid assessment. The second method involves utilizing the principle of time–temperature equivalence for creep test research [14,15,16,17,18,19]. This principle stipulates that creep deformation observed over a short period at a higher temperature is comparable to the deformation that occurs over a longer period at lower temperatures. By applying this principle, the long-term deformation properties of a material can be predicted in a significantly shorter timeframe. However, this method assumes that the creep mechanism of the material is the same at different temperatures. Moreover, temperature changes may lead to material aging or phase transitions, thereby introducing prediction uncertainties.

Beyond experimental methodologies, considerable effort has been devoted to the constitutive modeling of geosynthetic creep. Classical viscoelastic formulations, such as the Burgers model, combine Maxwell and Kelvin–Voigt elements to capture both primary and secondary creep stages, and they have been applied to geomembranes and geotextiles with varying degrees of success [20,21]. However, these models require the determination of multiple rheological parameters via the curve-fitting of experimental data, and their predictive accuracy often deteriorates when extrapolated beyond the calibration timescale. Power-law relationships, particularly the Findley model, offer a simpler empirical alternative and have been widely adopted for their mathematical tractability [22,23]. Nevertheless, power-law formulations may overestimate tertiary creep and may not adequately capture the long-term strain stabilization characteristics of reinforced geomembrane systems. Hyperbolic and other empirical functions provide additional fitting flexibility, but their parameters often lack clear physical meaning. Critically, the existing literature reveals a persistent fragmentation: most creep models have been developed and validated for a single material composition or a specific film thickness. A generalized predictive framework that systematically accounts for the influence of film thickness has not been reported.

In composite GM systems, the thickness of the polymeric membrane directly governs the cross-sectional load distribution and flexural rigidity of the composite. Under a constant tensile load, a thinner membrane experiences higher net section stress, which accelerates the development of viscoelastic and viscoplastic strain components [24]. Recent research has focused on evaluating the results of creep deformation using fast, accurate, and representative alternatives, particularly when examining long term effects [25,26,27,28,29,30]. The present study deliberately constrains the variable space to film thickness under a controlled stress ratio and constant environmental conditions, enabling a rigorous, mechanics-driven examination of this specific but practically significant structural parameter.

This study aims to reveal the quantitative variation in creep model parameters with film thickness through long-term creep tests on composite GMs of different thicknesses, and it aims to determine a representative time threshold for short-term data based on statistical convergence criteria. On this basis, an empirical model is constructed that only requires 72 h short-term constant-temperature creep test data to accurately predict the long-term creep behavior of composite GMs with different film thicknesses. The proposed model avoids the uncertainty in the mechanism due to temperature acceleration and provides a more direct, rapid, and reliable creep performance evaluation tool for engineering practice. This study not only deepens our understanding of the thickness–creep relationship in composite GMs but also provides a novel methodological reference for the long-term performance prediction of geosynthetic materials.

## 2. Materials and Methods

### 2.1. Test Materials

The specifications of the two types of geotextiles and one GM in the composite GM for seepage control projects are generally as follows: the base geotextile weighs 200–600 g/m^2^, and the GM thickness is 0.25–1.0 mm. These two typically used geotextiles and one GM in engineering were selected for the experiment research. The GM was a high-density polyethylene membrane, and the geotextile was a staple fiber needle-punched nonwoven fabric with a density of 200 g/m^2^. Following the test procedure specified in “Practice for testing geosynthetic materials” (SL/T235-2012) [31], the range of test variance *CV* was calculated to be 1.23% to 5.61%, which indicates a relatively low variability and meets the credibility requirements. The basic mechanical properties of composite GMs with different film thicknesses were measured using the YT010-5000 type geosynthetics universal testing machine produced by Zhejiang Wenzhou Jigao Testing Instrument Co., Ltd. of Wenzhou, China; the results are summarized in Table 1.

### 2.2. Test Methods

Owing to the varying mechanical properties of the composite GM in the longitudinal and transverse directions, specimens were cut in the longitudinal direction to ensure that the test results remain reliable and consistent with the actual application of the material. The specimen size was determined according to the wide-strip tensile method specified in “Practice for testing geosynthetic materials” (SL/T235-2012), resulting in a size of 200 mm × 100 mm. The findings from a previous experiment showed that the specimens are not damaged when the applied creep load is below 60% of their maximum breaking strength [24,32]. Because the maximum breaking strength of the composite GMs increases with film thickness, a load equivalent to 60% of the maximum breaking strength of the 0.4 mm composite GM was applied. This prevented specimen damage while still enabling the creep behavior of materials across varying thicknesses to be effectively evaluated.

**Figure 1:**
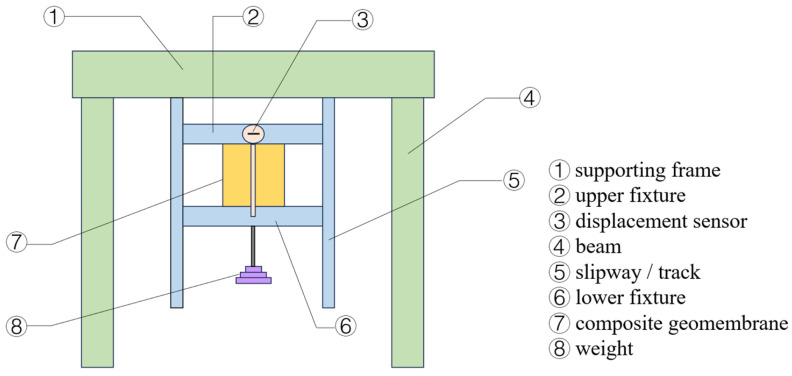
Schematic of tensile creep test.

The test was conducted using six independent instruments to ensure that the instruments did not interfere with one another. The temperature of the test environment was controlled at 20 ± 2 °C, with the relative humidity maintained at 60 ± 10%. A long-term creep test was conducted for 90 d in an indoor environment free from wind and other disturbances. Three parallel tests were configured simultaneously for each thickness level. To maintain load stability, weights were used to apply the load, with the GM thickness varying; however, the total mass of the applied weights remained constant. The weights were placed slowly and gently to minimize the impact of falling distance on the specimen. To avoid errors caused by manual readings, the deformation data were automatically collected using a large-range electronic dial gauge connected to a computer. A schematic of the tensile creep test is shown in Figure 1.

## 3. Test Result and Analysis

### 3.1. Creep Test Curve

**Figure 2:**
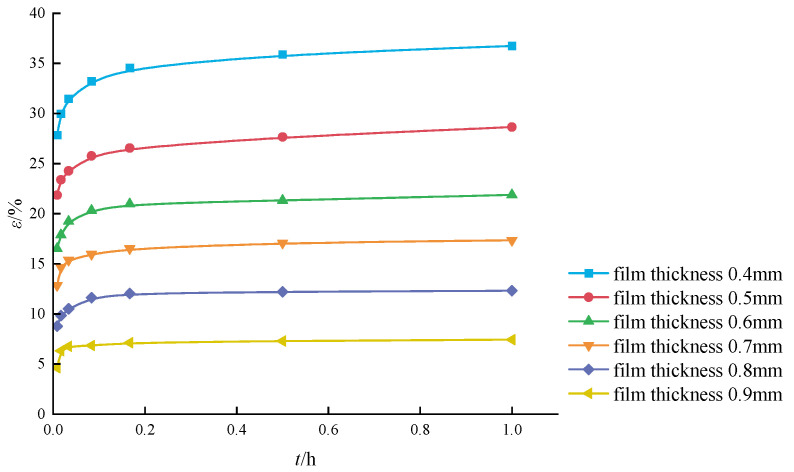
Creep curve within 1 h.

**Figure 3:**
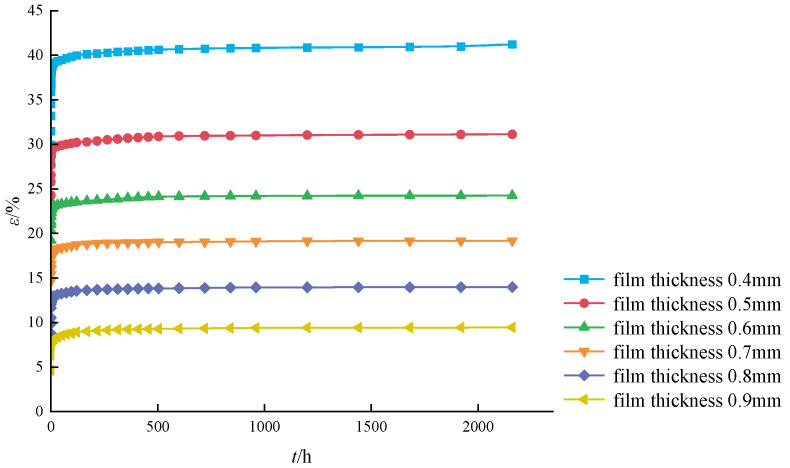
Creep curve over the 90-day period.

A 90-day experimental study was conducted on composite GMs of varying thicknesses under a constant load to investigate their creep behavior. The aim was to derive creep curves illustrating the strain–time relationship across different thicknesses. Figure 2 displays the creep curve for a 1 h period, while Figure 3 presents the creep curve over the entire 90-day test duration. While Figure 2 depicts the instantaneous primary creep behavior within the first hour, Figure 3 expands the view to illustrate the long-term creep evolution, clearly demarcating the transition from primary to secondary creep.

As shown in Figure 2 and Figure 3, the composite GMs of different thicknesses under the same tensile load exhibited deformation that aligned with the typical creep behavior of most materials, displaying the typical two-stage deformation characteristics of polymer materials. In Stage I, deformation rapidly developed within a relatively short period. Specifically, the specimen experienced a rapid increase in deformation immediately after the load was applied, and the strain changed significantly over time. During this process, the composite GM also showed characteristics of transverse shrinkage, which became more pronounced as the thickness of the GM decreased, indicating that this stage was primarily dominated by elastic deformation. Entering Stage II of stable deformation, the displacement gradually flattened with time, with the rate of increase continuously decreasing. This transition from primary to secondary creep, as well as the asymptotic approach to a stable strain, is characteristic of the logarithmic creep behavior identified in geosynthetics [9,23,24]. For the load level and duration of this test, no creep III stage was observed. The deformation of the composite GM grew linearly and smoothly, ultimately reaching a quasi-steady-state strain. As the thickness of the composite GMs decreased from 0.9 to 0.4 mm in steps of 0.1 mm, the creep strain at the end of the 90-day observation period reached approximately 9%, 14%, 20%, 24%, 31%, and 40%, respectively. Thinner specimens consistently exhibited higher strain than thicker ones. As thickness decreased, the creep strain increased more rapidly, resulting in a steeper curve. The initial strain increment increased as the thickness decreased, and the creep reached the steady-state stage after a longer time.

### 3.2. Film Thickness–Strain Relationship

**Figure 4:**
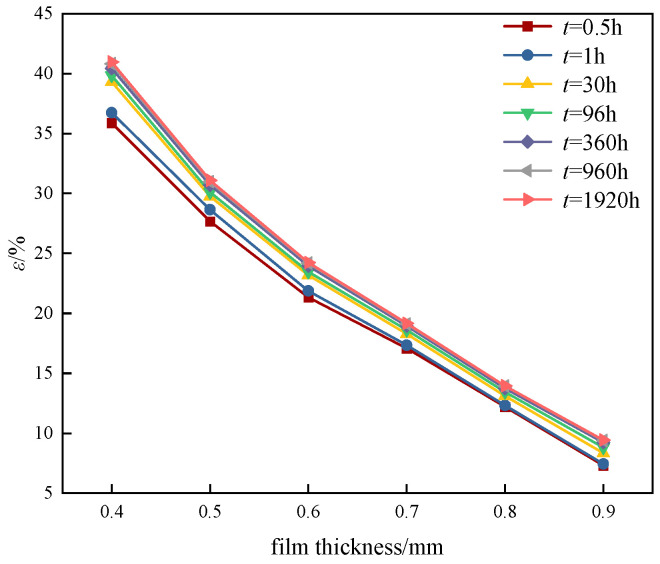
Isochronous film thickness–strain relationship.

To accurately analyze the effect of the time factor on different composite GM thicknesses, the film thickness–strain relationship at various time intervals was established, as shown in Figure 4.

Figure 4 indicates that the strain of the composite GM decreases with increasing GM thickness. There is an inverse relationship between film thickness and strain, and under each isochronous condition, the film thickness–strain curves are nearly coincident. After 0.5 h, the variation patterns of film thickness and strain remained consistent over time. As time increased, the strain changed only slightly, indicating that the relationship between film thickness and strain was strongly time-dependent. Meanwhile, as the GM thickness increased, the strain at final stabilization decreased gradually, with the final strain reducing by approximately 6% for every 0.1 mm increase in film thickness under a long-term stabilizing load.

## 4. Data Analysis

### 4.1. Model Establishment

**Figure 5:**
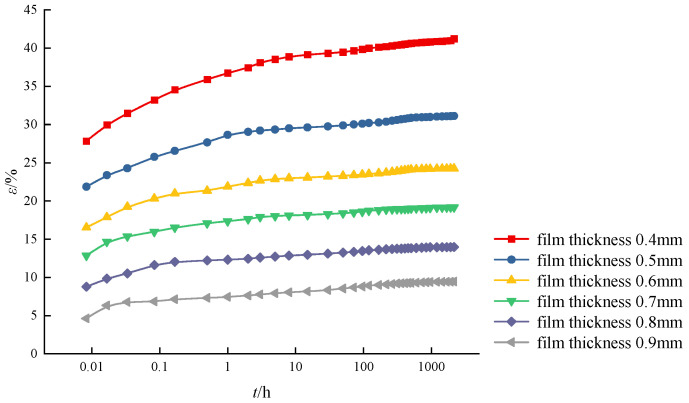
Creep curve fitted using the logarithmic function in Equation (1).

This empirical creep model offers several advantages. It often achieves a relatively ideal fitting effect with only the test parameters and is highly practical. It has a substantial application value in engineering practice [33,34,35,36]. Based on global research sources, the current empirical models used to predict the creep behavior of geosynthetics can be classified into four types: power and near-power, logarithmic and near-logarithmic, hyperbolic, and other empirical functions. Based on the creep test results for the composite GM, the strain increments under different thicknesses all proceeded in a decelerating manner, exhibiting decaying creep curves. The creep test data were fitted using different fitting models, and the fitting effects of each curve were analyzed. The results showed that the logarithmic function-type empirical creep model achieved the best fit for the data, whereas the correlation coefficients of other functions were relatively low, indicating poor fitting performance. Therefore, the logarithmic function-type model can effectively simulate the stress–strain relationship curves of composite GMs at different thicknesses and, to a certain extent, can be used to analyze the long-term deformation of composite GMs. Figure 5 shows the creep test data for 90 d; the pattern of the creep curve conforms to the characteristics of the logarithmic function.

Therefore, the long-term creep behavior of the composite GM can be expressed as a logarithmic function as

(1) y=aln(x)+b

where coefficient *a* represents the creep index, which indicates the slope of the deformation curve under a constant load and reflects the rate of creep development. The vertical line separating the primary and secondary creep stages is defined according to the parameter a in Equation (1), that is, the moment at which the tangent of the creep curve becomes constant. The coefficient *b* represents the initial strain value of the creep test, reflecting the instantaneous elastic deformation at the initial loading stage. Both *a* and *b* are related to film thickness. To establish a short-term model based on creep test data and reflect the characteristics of the various stages of creep, a relationship between the creep index and initial strain value under different film thicknesses must be constructed. Through the creep test, the corresponding logarithmic function creep index a and initial strain value b were obtained at different time points, as listed in Table 2.

**Figure 6:**
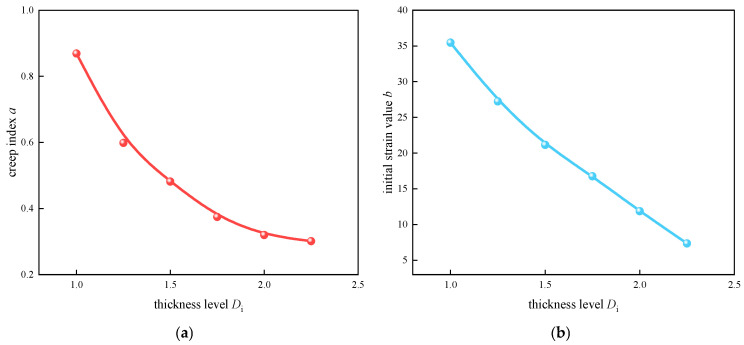
Relationship among creep index a, initial strain value b, and thickness level *Di*. (**a**) The relationship between creep index *a* and the thickness level; (**b**) The relationship between initial strain value *b* and the thickness level.

Figure 6 shows the relationships among the creep index *a*, initial strain value *b*, and thickness level *D_i_* (which is the ratio of each thickness level to the initial thickness). As the thickness level increased, both the creep index a and initial strain value b changed simultaneously, conforming to a quadratic function relationship and exhibiting a high degree of fitting accuracy. This indicates that the thickness of the geomembrane affects the value of each parameter in the logarithmic function. Therefore, based on the logarithmic function model, the functional relationship between the creep index a and initial strain value b at different thickness levels was established. Consequently, Equation (2) holds.

(2) $$\varepsilon =(cx^{2}+dx+e)\ln(x)+(fx^{2}+gx+h)$$

Based on the creep curve parameters in Table 2, substitution into Equation (1) yields the relevant material parameter values. The empirical creep model is

(3) $$s=6.66D_{i}^{2}-43.46D_{i}+71.8+(0.43D_{i}^{2}-1.83D_{i}+2.25)\mathrm{lg}t$$

It can be observed from Equation (3) that the creep index a and initial strain value b play a decisive role in the selection of the model parameters. Therefore, to establish a short-term creep model with better fitting, a statistical analysis was conducted on the creep index *a* and initial strain value *b*. The average value *M* and coefficient of variation *CV* of each parameter with respect to time are listed in Table 3.

In engineering statistics, a coefficient of variation below 10% is widely regarded as a reliable extrapolation indicator that data convergence is acceptable and sufficiently representative [13]. As presented in Table 3 and Table 4, the average values *M* and coefficient of variation *CV* of the creep index *a* changed significantly over time, whereas the average values *M* and coefficient of variation *CV* of the initial strain value *b* changed minimally over time. When the time exceeded 30 h, the coefficient of variation *CV* was below 15%, indicating that there was no significant difference between the creep index a and initial strain value *b*. When the time exceeded 72 h, the coefficients of variation *CV* were below 10%, indicating that the uncertainty in model parameters caused by insufficient observation time had been significantly reduced by the 72 h mark and that the data after 72 h exhibited good representativeness.

The creep data with a test time of 72 h can be fitted according to Equation (2), and the fitting formula within 72 h can be obtained as

(4) $$s=6.70D_{i}^{2}-43.84D_{i}+72.48+(0.51D_{i}^{2}-2.34D_{i}+3.04)\mathrm{lg}t$$

**Figure 7:**
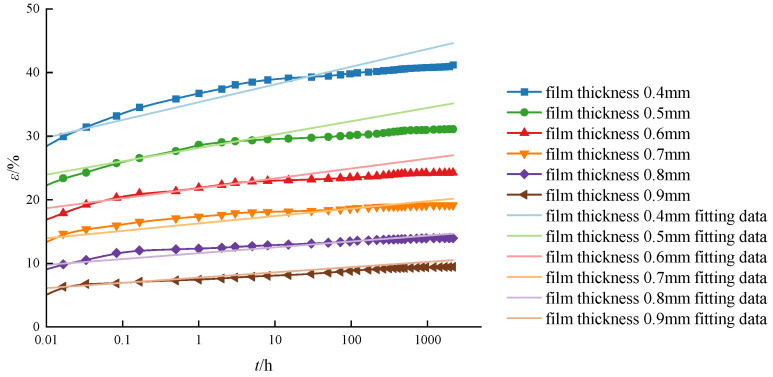
Relationship between creep fitting and measured values.

Using the parameters derived from the creep model based on the first 72 h of data, the creep behavior of six composite GMs with different thicknesses was modeled and compared with the measured values. As shown in Figure 7, the predicted curve was at the same level as the measured curve.

### 4.2. Statistical Analysis

To systematically evaluate the predictive accuracy and reliability of the established short-term creep prediction model, four complementary statistical metrics were employed: the Pearson correlation coefficient, root mean square error (RMSE), mean absolute error (MAE), and mean absolute percentage error (MAPE). A comparative analysis was conducted between the experimentally measured strain values from the 90-day long-term creep tests of composite GMs with varying film thicknesses and the fitted values obtained from two models: the thickness-dependent logarithmic function model fitted over the entire test period and the proposed short-term prediction model calibrated using only the first 72 h of data. The specific calculation results are summarized in Table 5.

As presented in Table 5, the Pearson correlation coefficients for both models exceeded 0.9 across all film thickness levels, indicating an extremely strong positive linear correlation between the model outputs and the experimentally measured values. Regarding the error metrics, the RMSE, MAE, and MAPE values for the thickness-dependent logarithmic function model ranged from 0.48% to 1.18%, 0.41% to 0.97%, and 0.03 to 0.05, respectively. In comparison, the corresponding values for the short-term prediction model were 0.49% to 2.45%, 0.39% to 2.09%, and 0.03 to 0.07, respectively. These statistical findings collectively validate that when the coefficients of variation of the model parameters decreased to below 10% after 72 h of testing, the short-term data exhibited sufficient statistical representativeness and could provide a reliable prediction of the long-term creep behavior of the material under constant room-temperature loading.

## 5. Conclusions

A 90-day long-term creep test was conducted on composite GMs of different thicknesses; their corresponding creep behaviors were compared and analyzed. A short-term prediction model for the creep behavior of composite GMs with different thicknesses was established. The main conclusions are as follows:

(1) Under the applied constant load, the creep strain of the composite GM exhibited an inverse relationship with film thickness. As the film thickness increased from 0.4 to 0.9 mm, the final stabilized strain decreased from approximately 40% to 9%, corresponding to an average reduction of approximately 6% per 0.1 mm increase in thickness. This relationship remained consistent across different time intervals, indicating a strongly thickness-dependent strain response.
(2) The long-term creep behavior can be adequately described by a thickness-dependent logarithmic function model, in which both the creep index *a* and the initial strain value *b* vary with the dimensionless thickness level *D_i_* according to quadratic polynomial functions, yielding high fitting accuracy. This confirms that film thickness significantly influences the parameter values of the logarithmic creep model.
(3) Statistical analysis of the model parameters revealed that after 72 h of testing, the coefficients of variation for both the creep index *a* and the initial strain *b* were below 10%, indicating that the short-term dataset achieved acceptable statistical stability. The short-term creep model established on this basis yielded Pearson correlation coefficients ranging from 0.94 to 0.97 with the 90-day measured values, as well as RMSEs between 0.49% and 2.45% and MAPEs between 0.03 and 0.07. Therefore, within the experimental framework of this study, 72 h data enabled a reasonable extrapolation of creep strain up to 90 days.

The short-term creep prediction model proposed herein effectively predicts the long-term creep behavior of composite GMs with different thicknesses using only 72 h of constant-temperature creep test data, thereby significantly reducing the testing period. It provides a practical and reliable tool for rapidly assessing the creep performance of composite GMs in engineering practice. Nevertheless, several limitations should be acknowledged. First, the experimental program was restricted to a single type of composite GM and a specific range of film thicknesses. Second, the tests were conducted under idealized laboratory conditions that did not include field-relevant environmental factors such as ultraviolet radiation, thermal cycling, chemical exposure, or fluctuating temperature and humidity. Third, although the 90-day creep duration covers the primary and secondary creep stages, it does not capture tertiary creep or long-term rupture behavior that may occur over a service life of several decades. Therefore, future work should extend the present methodology to a broader range of geomembrane materials and incorporate multi-factor accelerated testing that accounts for the coupled effects of temperature, humidity, and UV exposure. Moreover, the model should be validated against field data or longer-duration laboratory creep tests to assess its performance in predicting creep behavior over engineering-relevant service lives. Such efforts will help transform the current statistical convergence-based approach into a more general and practically robust design tool.

## Figures and Tables

**Table 1:** Technical index of the composite GM.

Film Thickness (mm)	Rupture Strength (kN/m)		Elongation at Break (%)		CBR Burst Strength (kN)	Tear Strength (kN)	
	Longitudinal	Transverse	Longitudinal	Transverse		Longitudinal	Transverse
0.4	14.4	14.6	51	51	2.7	0.50	0.51
0.5	16.3	16.9	56	53	3.1	0.57	0.59
0.6	18.1	18.4	66	66	3.6	0.64	0.64
0.7	21.3	21.6	69	67	4.4	0.71	0.74
0.8	23.9	24.2	73	69	5.2	0.79	0.80
0.9	26.8	27.3	88	92	6.9	0.91	0.92

**Table 2:** Creep curve parameters at different times.

*T*		Different Film Thickness (mm)											
		0.4		0.5		0.6		0.7		0.8		0.9	
		*a*	*b*	*a*	*b*	*a*	*b*	*a*	*b*	*a*	*b*	*a*	*b*
min	5	2.30	39.12	1.66	29.94	1.67	24.65	1.30	19.50	0.12	14.66	0.91	9.49
	10	2.18	38.64	1.55	29.49	1.49	23.92	1.13	18.81	1.09	14.17	0.71	8.70
	30	1.95	37.76	1.40	28.95	1.19	22.81	0.95	18.14	0.86	13.31	0.54	8.06
h	1	1.81	37.28	1.36	28.81	1.07	22.37	0.84	17.77	0.73	12.85	0.46	7.77
	2	1.70	36.91	1.29	28.57	0.99	22.12	0.77	17.52	0.64	12.54	0.41	7.61
	5	1.59	36.58	1.16	28.19	0.91	21.86	0.70	17.30	0.55	12.26	0.37	7.49
	15	1.54	36.43	1.10	28.02	0.87	21.74	0.66	17.20	0.52	12.18	0.36	7.46
	30	1.38	36.07	0.96	27.70	0.76	21.50	0.58	17.01	0.46	12.04	0.34	7.40
	72	1.24	35.80	0.85	27.49	0.68	21.35	0.52	16.90	0.42	11.97	0.33	7.38
d	5	1.16	35.66	0.79	27.38	0.63	21.26	0.49	16.85	0.40	11.93	0.32	7.38
	9	1.09	35.58	0.74	27.32	0.59	21.21	0.46	16.82	0.38	11.91	0.32	7.38
	15	1.02	35.50	0.69	27.27	0.56	21.18	0.44	16.79	0.36	11.89	0.32	7.38
	25	0.96	35.46	0.66	27.24	0.53	21.16	0.41	16.77	0.35	11.88	0.32	7.37
	40	0.93	35.45	0.64	27.23	0.51	21.15	0.40	16.77	0.34	11.88	0.31	7.37
	80	0.88	35.45	0.61	27.24	0.49	21.15	0.38	16.77	0.32	11.88	0.31	7.37

**Table 3:** Statistical analysis of the variation in creep index *a* over time.

*T*		Different Film Thickness (mm)											
		0.4		0.5		0.6		0.7		0.8		0.9	
		*M*	*CV* (%)	*M*	*CV* (%)	*M*	*CV* (%)	*M*	*CV* (%)	*M*	*CV* (%)	*M*	*CV* (%)
min	5	1.45	31	1.03	33	0.86	41	0.67	41	0.50	47	0.42	40
	10	1.39	29	0.99	31	0.81	36	0.62	35	0.53	41	0.39	28
	30	1.33	26	0.94	29	0.75	30	0.58	30	0.49	33	0.36	18
h	1	1.28	24	0.90	28	0.72	27	0.55	27	0.46	27	0.35	13
	2	1.23	22	0.86	26	0.68	24	0.53	24	0.43	22	0.34	9
	5	1.18	20	0.82	23	0.65	22	0.50	21	0.41	18	0.33	6
	15	1.13	18	0.78	20	0.62	19	0.48	18	0.39	15	0.33	5
	30	1.08	15	0.74	15	0.59	15	0.46	14	0.38	11	0.32	3
	72	1.04	12	0.71	11	0.57	11	0.44	11	0.37	9	0.32	2
d	5	1.01	9	0.69	9	0.55	9	0.43	9	0.36	7	0.32	1
	9	0.98	7	0.67	7	0.54	7	0.42	7	0.35	6	0.32	2
	15	0.95	5	0.65	4	0.52	5	0.41	5	0.34	4	0.32	2
	25	0.92	4	0.64	3	0.51	3	0.40	3	0.34	4	0.31	2
	40	0.91	3	0.63	2	0.50	2	0.39	3	0.33	3	0.31	0
	80	0.88	0	0.61	0	0.49	0	0.38	0	0.32	0	0.31	0

**Table 4:** Statistical analysis of the variation in initial strain value *b* over time.

*T*		Different Film Thickness (mm)											
		0.4		0.5		0.6		0.7		0.8		0.9	
		*M*	*CV* (%)	*M*	*CV* (%)	*M*	*CV* (%)	*M*	*CV* (%)	*M*	*CV* (%)	*M*	*CV* (%)
min	5	36.51	3	28.06	3	21.96	5	17.39	5	12.49	7	7.71	8
	10	36.33	3	27.92	3	21.77	4	17.24	3	12.34	5	7.58	5
	30	36.15	2	27.80	2	21.60	2	17.12	2	12.19	4	7.49	3
h	1	36.01	2	27.71	2	21.50	2	17.04	2	12.10	2	7.45	2
	2	35.90	1	27.60	2	21.43	2	16.97	1	12.03	2	7.42	1
	5	35.80	1	27.51	1	21.36	1	16.92	1	11.98	1	7.40	1
	15	35.71	1	27.43	1	21.30	1	16.88	1	11.95	1	7.39	0
	30	35.62	1	27.36	1	21.25	1	16.84	0	11.92	0	7.38	0
	72	35.56	0	27.31	0	21.21	0	16.81	0	11.91	0	7.38	0
d	5	35.52	0	27.28	0	21.19	0	16.80	0	11.90	0	7.38	0
	9	35.49	0	27.26	0	21.17	0	16.78	0	11.89	0	7.37	0
	15	35.47	0	27.25	0	21.16	0	16.78	0	11.88	0	7.37	0
	25	35.45	0	27.24	0	21.15	0	16.77	0	11.88	0	7.37	0
	40	35.45	0	27.24	0	21.15	0	16.77	0	11.88	0	7.37	0
	80	35.45	0	27.24	0	21.15	0	16.77	0	11.88	0	7.37	0

**Table 5:** Comparison of statistical evaluation metrics between measured and model-predicted creep values of composite GMs with different thicknesses.

Film Thickness (mm)		0.4	0.5	0.6	0.7	0.8	0.9
Improved logarithmic function model	RMSE (%)	1.18	1.13	0.77	0.82	0.55	0.48
	MAE (%)	0.92	0.97	0.58	0.74	0.48	0.41
	MAPE	0.03	0.03	0.03	0.04	0.04	0.05
	Pearson Correlation Coefficient	0.95	0.94	0.94	0.94	0.95	0.97
Short-term prediction model	RMSE (%)	1.97	2.45	1.64	0.69	0.49	0.67
	MAE (%)	1.74	2.09	1.39	0.59	0.39	0.60
	MAPE	0.04	0.07	0.06	0.03	0.03	0.07
	Pearson Correlation Coefficient	0.95	0.94	0.94	0.94	0.95	0.97

## Data Availability

The original contributions presented in the study are included in the article, further inquiries can be directed to the corresponding author.
